# Establishment of pediatric developmental dysplasia of the hip biobank: Shanghai children’s hospital experience

**DOI:** 10.1007/s10561-022-09995-3

**Published:** 2022-02-25

**Authors:** Dan Yang, Shiqi Wang, Chenghui Ke, Qichao Ma, Lingyan Fan, Yichen Wang, Mengjie Chen, Hao Ying, Sun Wang, Qin Jiao, Yang Shen, Lihua Zhao

**Affiliations:** 1 Department of Orthopedics, Shanghai Children’s Hospital, Shanghai Jiao Tong University, 355 Lane Luding Rd, Putuo District, Shanghai, 200062 People’s Republic of China; 2 NHC Key Laboratory of Medical Embryogenesis and Developmental Molecular Biology & Shanghai Key Laboratory of Embryo and Reproduction Engineering, Shanghai, 200040 People’s Republic of China

**Keywords:** Developmental dysplasia of the hip, Pediatric patient, Biobank, Database

## Abstract

Developmental dysplasia of the hip (DDH) is a debilitating condition that affects 1–7% of newborns. Children with DDH, not treated early and effectively, will easily lead to disability. A better understanding of the biology of DDH is critical to the development of prognostic biomarkers and novel therapies. The purpose of this study was to establish a biobank of DDH genetic resources, to facilitate clinical and basic scientific research. The biological specimen and clinical data of DDH were collected in Shanghai Children’s Hospital from 2014 to 2021. The collection of blood samples was performed at definitive diagnosis and review, tissue specimens were performed at definitive surgery. The clinical data was collected at the whole stage of DDH patients at diagnosis, treatment and follow-up. A total of 528 patients with DDH were enrolled in this study, 90 were men and 438 were women, with the mean age of 4.67 years. The numbers of tissue and blood specimens reached 2172 and 1490, respectively. The quality test results showed that the DNA concentration decreased slightly with the extension of storage time, but the DNA purity did not change. Meanwhile, the extension of storage time slightly affected the stability of protein of tissue samples but did not affect the expression of the housekeeping gene. The DDH biobank built has the potential of monitoring disease pathogenesis and progress, which could provide specimens to the researchers improving the biological understanding and provide guidance of clinical treatment of this disease to clinicians.

## Introduction

Developmental dysplasia of the hip (DDH) is the most common malformation of hip joint in pediatric orthopedics (Pollet et al. 2017; Feldman et al. 2019). The rate of DDH in the neonatal period of North Central Europe (0–28 days) ranges from 1 to 7% (Woodacre et al. 2016). In England, the incidence rate of DDH was 1.28 per 1000 live births (Broadhurst et al. 2019). In China, the average incidence rate of DDH varies from 1 to 10‰, but the incidence rate varies significantly among different races and regions, which is related to genetic factors, environmental impacts, and lifestyle (Pollet et al. 2017). Children with DDH, but not timely implementation of appropriate treatment, will suffer from early-onset osteoarthritis (OA) of the hip, which is easy to lead to disability in severe cases (Tetsunaga et al. 2017). Owing to its high incidence rate and high disability rate, the early screening and treatment of DDH has become the focus and points in pediatric orthopedic and child health care departments (Azzopardi et al. 2011; Shaw et al. 2016a, b). A better understanding of the biology of DDH need to screening and diagnosis early and identify accurate molecular markers and improve patient outcome. Thus, important equipment for understanding the biology and genetic characteristics of DDH is access to patient materials. However, there is no DDH biobank reported in the literature in the world.

In the 1990s, screening of DDH was listed as one of the important items of routine physical examination for infants and young children in Germany, Britain, North America, and other European and American countries (Stein-Zamir et al. 2008). In Britain, Austria and other European countries, screening of DDH was also protected by legal system (Stein-Zamir et al. 2008). In China's first-tier cities such as Shanghai, Beijing and Tianjin, enough attention has also been paid to the early screening of DDH. However, in remote cities such as Tibet, Yunnan and Guizhou, the screening rate and diagnosis and treatment rate of DDH are still low (Li et al. 2013; Engesaeter et al. 2011). The awareness of families in these areas is even lower (Engesaeter et al. 2011). So, in the past years, we screened DDH patients in Shigatse (Tibet) and the eastern, central, and western regions of China, and obtained many clinical data such as epidemiology and imaging. We found that the prevalence of DDH in Shigatse, Tibet was approximately 174.9/1000 infants (106/606) (Zhao et al. 2019), much higher than that in other parts of China. At the same time, we have set up a medical team to follow up Tibet regularly every year to help Tibet establish a DDH early screening, diagnosis and treatment medical team. Therefore, the public welfare project of “gesanghua’s love” has been formed. The above work helped us establish an early DDH clinical data biobank.

DDH is a complex disorder, the etiology of DDH includes both genetic factors and environmental factors (Zhao et al. 2019; Duman et al. 2019). Environmental risk factors include breech presentation, oligohydramnios, primiparity and so on (Chan et al. 1997; Stein-Zamir et al. 2008). Epidemiological studies showed that 67.88% of DDH cases were genetically related (Ceylaner et al. 2008). Some genetic studies had shown that DDH was consistent with some characteristics of autosomal dominant inheritance, but it does not show a simple Mendelian inheritance model (Shi et al. 2012). Maybe because of its complex etiology, DDH often shows incomplete penetrance (Feldman et al. 2013). In a previous study on a large multi-generation family, we identified a variant in bone morphogenetic proteins-2-inducible kinase (*BMP2K*) known to affect bone metabolism (Zhao et al. 2017) and a nonsense variant in *PTGFR* (Wang et al. 2018). This termination codon variant in exon 3 of *PTGFR* gene (c.c922t: p.r308x) located on chromosome 1 may be the pathogenic variant of DDH (Zhao et al. 2017). Although evidence for the genetic cause of DDH has been widely studied and obtained many results, the exact pathogenesis, pathological and molecular mechanisms of DDH remains unclear. Moreover, the incidence of DDH varies greatly in different regions of China. These have caused great deviations to the basic research work. Therefore, the establishment of a multi-component database of patients’ samples of DDH (blood, tissues, and DNA) can effectively change these circumstances, and provide a guarantee for obtaining large research samples in a short time and exploring appropriate treatment strategies based on the natural and original biological characteristics of DDH.

Taken together, the objective of the current study is to establish a biobank of DDH with multi-component samples, to facilitate clinical and basic scientific research. Although the establishment of China's Standardization Organization database has just started, we believe our work will be conducive to standardized DDH biobank establishment. Our biobank was supported by the Orthopedic Department of Shanghai Children’s Hospital affiliated to Shanghai Jiao Tong University, one of the DDH specialized disease treatment centers in China. The DDH biobank we have established is a large sample size biobank publicly reported in China, which will provide valuable information and sample material support for clinical treatment and basic research of DDH.

## Materials and methods

Clinical samples of DDH patients were obtained from Shanghai Children’s Hospital Affiliated to Shanghai Jiao Tong University. All samples were collected with the donor being informed completely and with their consent. The procedures were approved by the Institutional Ethical Review Board of the Shanghai Children’s Hospital (2020R043-E01).

### Subjects and clinical analysis

Results of clinical exams and radiograph imaging of the hips were evaluated by three pediatric orthopedic surgeons, with clinical opinions of two additional surgeons elicited in any case of disagreement. Radiographic measurements of the hip were taken and affected individuals were identified according to the following criteria: Perkin quadrant (the femoral head is not in the inner lower Perkin quadrant), Acetabular index (> 25°), Shenton’s line (disrupted) and center edge angle (< 20°).

### Specimen origins

The collection of the specimens began in 2014. The blood samples were collected at definitive diagnosis and review. The tissue specimens were obtained at the definitive surgery. The clinical data was collected at the whole stage of DDH patients at diagnosis, treatment and follow-up. All data and samples were obtained with the patient's informed consent.

### Collection of blood samples

**Fig. 1:**
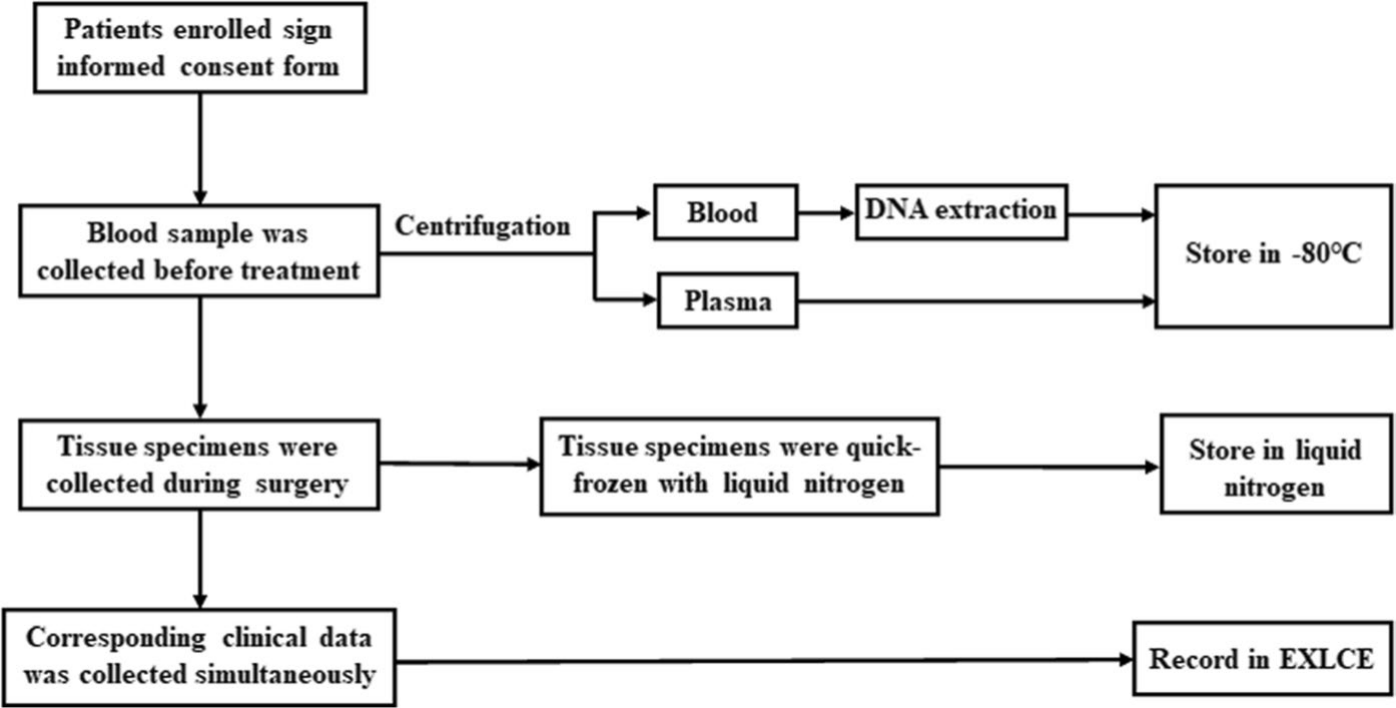
Workflow of the Collection of blood samples, tissue specimens and clinical data

The collection of blood samples was performed at definitive diagnosis and review. 3 mL of the blood sample was collected to the purple top blood collection tube before treatment, and was centrifuged at 3000 g × 10 min to separate plasma and blood cells. Some blood cells are used to extract DNA following the manufacturer's guidelines. Sub package blood cells, DNA, and plasma into small volumes separately and put into tubes, and then store at −80 °C. The workflow of specimens’ collection was shown in Fig. 1.

### Collection of tissue specimens

The collection of tissue specimens was performed at definitive surgery. After the surgery schedule was settled, technicians responsible for the specimen collection would be informed. Surgical treatment of DDH patients requires osteotomy. The resected tissue samples, including round ligament, transverse ligament or joint capsule, were firstly examined visually by surgeons, and then the tissue samples were handed over to our technicians for collection. The specimens were cut into small pieces of 1 cm^3^. A piece in each part is put into cryopreservation tube and then put into the liquid nitrogen tank for quick freezing. The lag between specimen removal and storage should not exceed 30 min. The workflow of specimens’ collection was shown in Fig. 1.

### Collection of clinical data

Clinical data were extracted from the electronic databases of Shanghai Children’s Hospital. Most of the stored specimens have corresponding clinical data, including age at enrollment; sex and race; Family history; time to last follow-up. Follow-up was performed every 6 months via outpatient or telephone, up to 3 years. An Excel database was established to record this information and managed by our technicians who received professional training.

The X-ray imaging of DDH patients were performed in parallel with the blood sample collection mentioned above. These image data were also collected in our database, corresponding to tissue specimens.

### Sample borrowing process

The borrowers are asked to fill out a request form that includes the following items: the name and institution of the applicant and project leader, the number and type of samples, description of the research project and experiments to be performed. After the submission, our biobank and research office administrators and chief investigator will review the application and sign it. The samples and data must be used only for research as specified in the previous request form and the remaining samples must be returned to our biobank in time. The patient privacy will be protected throughout the whole process.

### DNA extraction and the concentration and purity detection

The DNA was extracted from blood cell specimens using the DNeasy Blood Kit (Qiagen, German) following the manufacturer's guidelines. The concentration and purity of DNA extracted from blood were detected by Nanodrop 2000 (Thermo Scientific, USA). Polymerase chain reaction products of β-Actin were amplified with specific primers (Act-F1: atcatgtttgagaccttcaacacc, Act-R1: ccaggaaggaaggctggaagagtg; Act-F2: ctgagcgcaagtactccgtgtgga, Act-R2: ttacacgaaagcaatgctatcacc) and the primers were synthesized by Shanghai Major bio-Co., Ltd.

### Protein extraction and housekeeping gene β-Actin expression detection

The whole protein was extracted by RIPA Lysis Buffer (Beyotime Biotechnology, China), and the concentration was detected by BCA protein assay kit (Beyotime Biotechnology, China). Cell lysates were kept on ice for 30 min and centrifuged at 16,000 g for 3–5 min at 4 °C. Supernatants were collected and boiled in 5 × SDS loading buffer, the same amounts of protein were separated by 10% SDS-PAGE and stained by Coomassie or blotted onto polyvinylidene fluoride membranes (Millipore, USA). The primary antibody used in the experiment was β-Actin mouse monoclonal antibody (ab8226, Abcam, USA).

## Results

### Specimen and clinical data collection

A total of 528 patients with DDH were enrolled in this study from 2014 to 2021. Of these patients, 90 were men and 438 were women. The mean age at diagnosis of DDH was 4.67 years. Among these patients, Tibetans accounted for the majority up to 400, and the rest were Han patients up to 128. The number of DNA was 892. The numbers of tissue and blood specimens reached 2172 and 1490, respectively. The corresponding clinical and demographic data were collected in our electronic database. The characteristics of the biobank are shown in Table 1.

**Table 1:** Characteristics of the DDH Biobank

Features	Number
Patients	528
Age	
Minimum	0.25
Maximum	15
Mean	4.67
Sex	
Male	90
Female	438
Race	
Han	128
Tibetan	400
Specimens	
Blood	1490
Tissue	2172
DNA	892

**Fig. 2:**
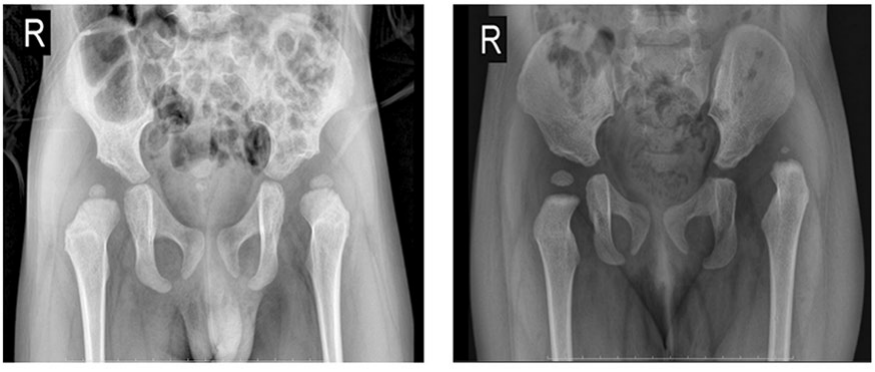
Representative pictures of DDH patients

Both clinical data and X-ray imaging data of DDH patients were performed in parallel with the specimen sample collection mentioned above. These image data were also collected in our database, corresponding to tissue specimens (Fig. 2).

### DNA extraction and the concentration and purity detection

The stored DNA samples were subpackaged into small volumes and put into tubes, so the number reached 892. The DNA sequencing was performed on these samples to observe the genomic alteration of DDH.

**Fig. 3:**
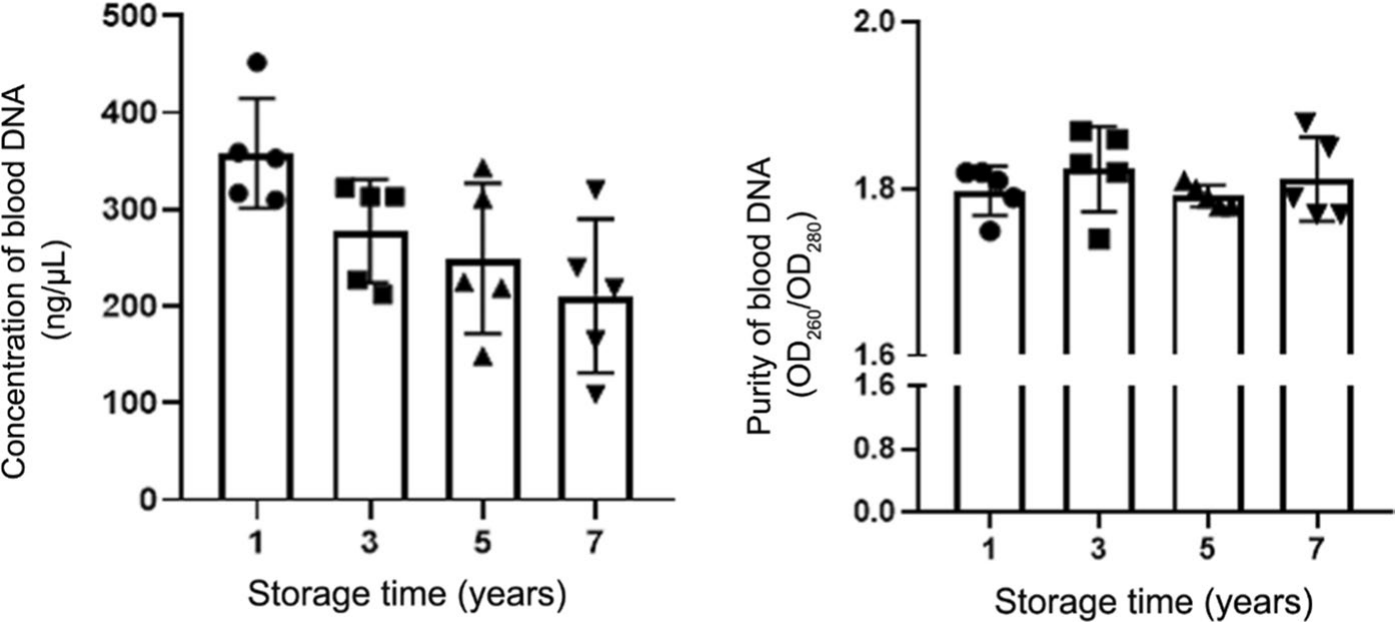
Concentration of DNA extracted from blood at different storage years

**Fig. 4:**
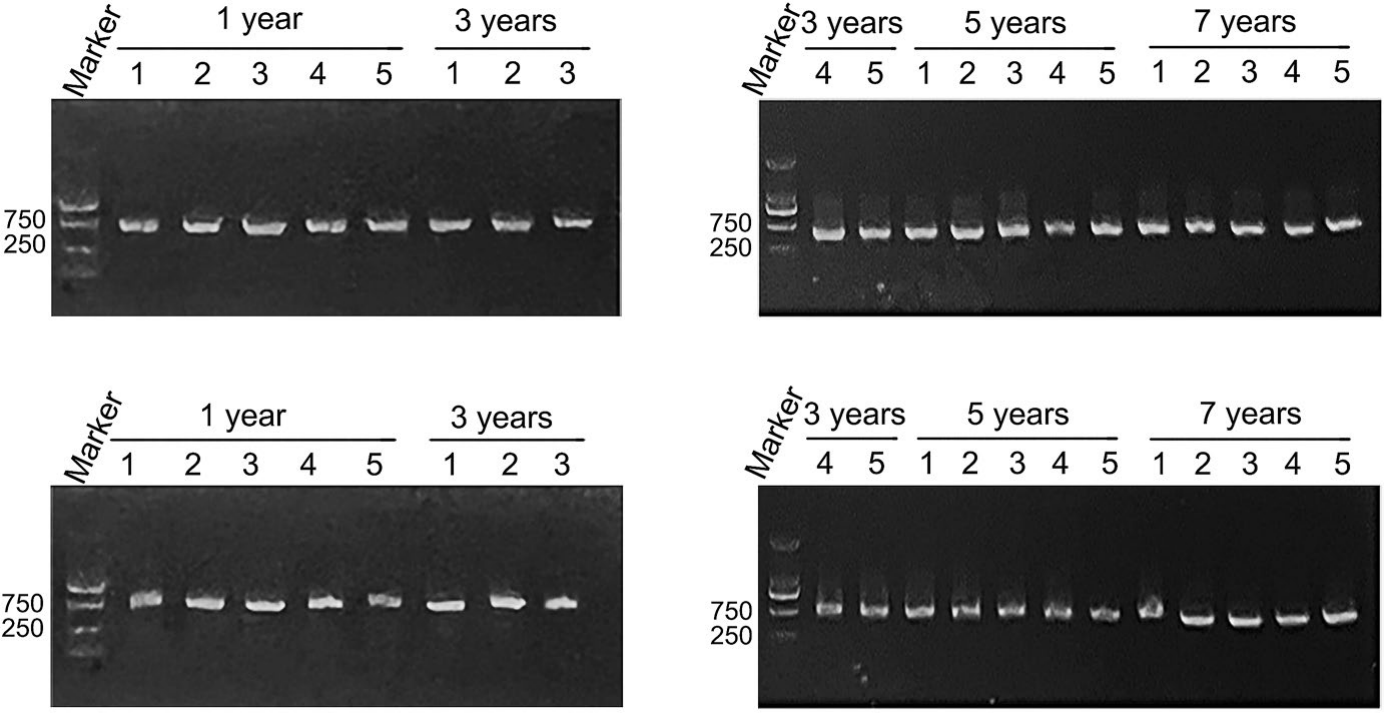
Nucleic acid gel electrophoresis of β-Actin amplified from blood DNA

To prove that the samples stored for many years are still valid and available, we selected blood samples from different storage times (1, 3, 5 and 7 years), to extracted DNA and detect their concentration and purity. There are 20 blood samples out of the biobank, with an average storage time of 5 samples. The concentration and purity of DNA extracted from blood were detected by Nanodrop 2000 (Thermo Scientific, USA). We found that the storage time did not affect the purity of DNA, but the concentration of DNA slightly decreased with the extension of storage time (Table 2, Fig. 3). The results of nucleic acid gel electrophoresis showed that the DNA samples of different storage times were intact and unbroken (Fig. 4).

**Table 2:** Concentration and purity of DNA extracted from blood at different storage years

Storage time (Years)	Concentration (ng/µL)	Purity (OD_260_/OD_280_)
1	358.10	1.80 ± 0.03
3	277.68	1.82 ± 0.06
5	249.28	1.80 ± 0.01
7	210.64	1.81 ± 0.05

### Protein extraction and housekeeping gene β-Actin expression detection

**Fig. 5:**
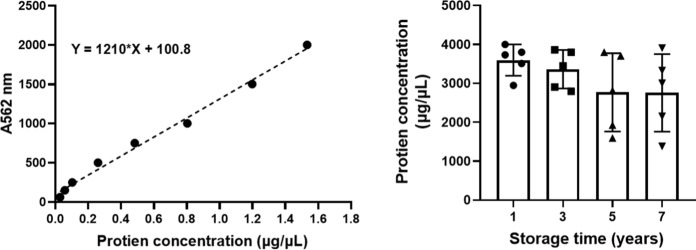
Concentration of protein extracted from tissues at different storage years

**Fig. 6:**
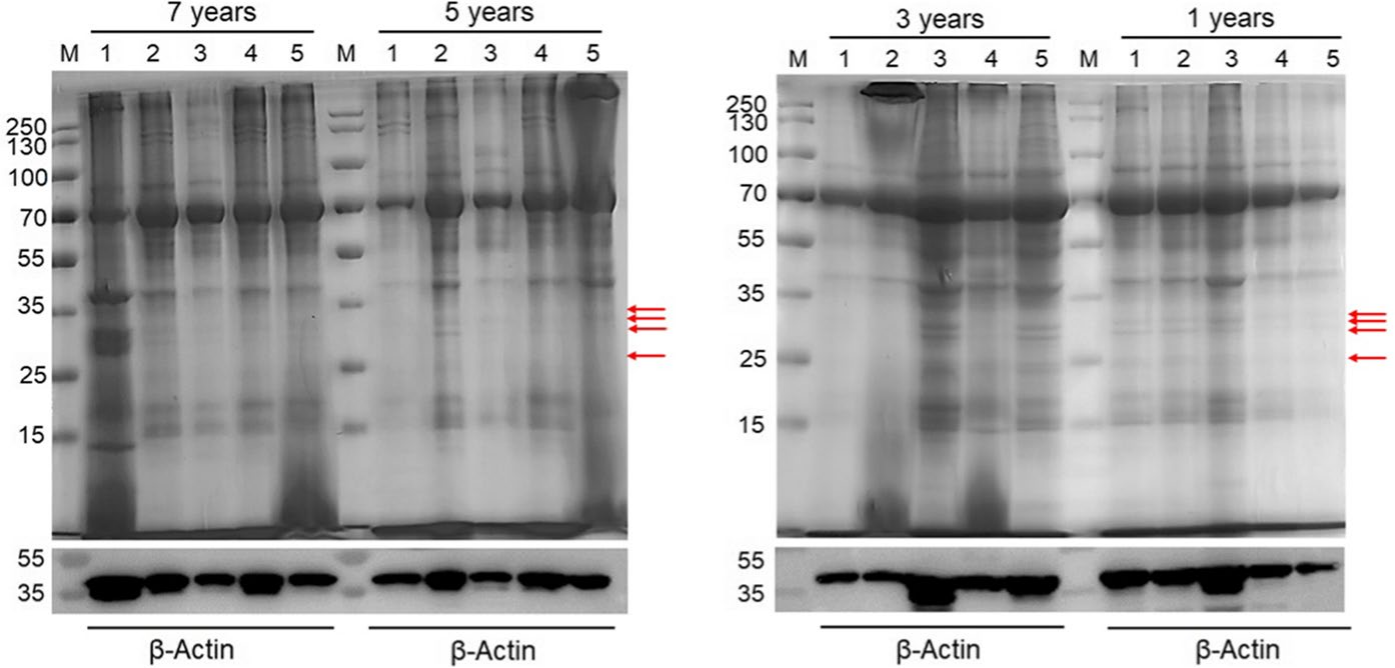
Expression of β-Actin extracted from tissues at different storage time

To prove that the stored tissue samples can be used for protein experimental research, we selected the samples stored at different times and extracted the total protein. There are 20 tissue samples out of the biobank from different storage times (1, 3, 5 and 7 years). We found that the concentration of protein slightly decreased with the extension of storage time (Fig. 5). We also detected the expression of housekeeping gene β-Actin of protein samples at different storage times. The result showed that the storage time did not remarkedly affect the expression of β-actin (Fig. 6). However, the result of SDS-PAGE showed that some bands stained with Coomassie bright blue in protein samples stored for 1 and 3 years disappeared in protein samples stored for 5 and 7 years, as shown by the red arrow in Fig. 6. The above results indicated that with the extension of storage time, it would affect the stability of protein in tissue samples, but had no effect on the expression of housekeeping gene.

### Correlative studies and published manuscripts

The purpose of our DDH biobank is to facilitate clinical and basic scientific research. Therefore, the quality of this biobank could be evaluated by correlative studies and the resulting publications that included data from our biobank. Table 3 lists the published manuscripts that have used these samples. These studies have contributed to the discovery of more biological characteristics of DDH. These samples in our biobank have played an important role in these studies and contributed to the advance in clinical and basic scientific research of DDH.

**Table 3:** Published manuscript that have specimens in our DDH biobank

Author (year)	Title	Journal
Zhao et al. (2019)	Screening for developmental dysplasia of the hip in infants in tibet identifies increased prevalence associated with altitude	Medical Science Monitor
Wang et al. (2018)	Whole genome exon sequencing of developmental dislocation of the hip in a chinese family	Chinese Journal of Pediatric Surgery
Zhao et al. (2017)	Expression of PTGFR gene in articular capsule and round ligament if children with DDH and its correlation with THE pathogenesis of DDH	Chongqing Medicine
Zhao et al. (2017)	A recurrent mutation in bone morphogenic proteins-2-inducible kinase gene is asscociated with devolpmental dysplasia of the hip	Experimental and Therapeutic Medicine

## Discussion

DDH is the most common malformation of the hip joint in pediatric orthopedics. Children with DDH, not treated early and effectively, will easily lead to disability. A better understanding of the biology of DDH is critical to the development of prognostic biomarkers and novel therapies. Nevertheless, there is still no reported biobank of DDH in the world. Several biobanks of bone-related diseases including bone health, bone quality and fracture, rheumatoid arthritis, osteoporosis, and so on, have been created worldwide (Darling et al. 2018; Kringelbach et al. 2016; Zahra et al. 2021). However, these studies mainly focus the light on the collection of clinical data not biospecimen. The existing research has not formed a unified standard for the construction of biobank, and the guidance for the establishment of DDH biobank is still lacking. Thus, we established a DDH biobank with multi-component samples including biological specimens and clinical data, to facilitate clinical and basic scientific research.

In the collection of the biobank from 2014 to 2021, 528 patients were included and the corresponding clinical data were obtained in the eastern, central, and western regions of China. This biobank will help to clarify the incidence rate and difference of DDH in different regions of China. The etiology of DDH is complicated and has a close relationship with environment and lifestyle (Palomäki et al. 2020; Harcke et al. 1999; Hatzikotoulas et al. 2018). Therefore, the incidence rate of DDH is obviously different in different ears of China. By analyzing the clinical information of DDH patients screened, we found that among all 528 DDH patients, 400 Tibetans (75.76%) and 128 Han people (24.24%). This result showed that remote area such as Tibet is more likely to induce DDH due to altitude, climate, horse herding, infant swaddling, low medical level, and so on. Additionally, 528 patients included 438 women (82.95%) and 90 men (17.05%). DDH presents high prevalence in Asians, and females are more frequently involved with a sex ratio of 4–10:1 (Li et al. 2017), female gender is the most important risk factor for DDH (Li et al. 2017; Ortiz et al. 2012; Talbot et al. 2013; Shaw et al. 2016a, b). Laxity induced by maternal hormones and limited in utero hip mobility also are the leading causes. In infants with DDH, abnormally increased laxity of the hip capsule and surrounding ligaments have been attributed to the effects of maternal hormone relaxin and a higher concentration of estrogen receptors (Desteli et al. 2013).

Besides environment and lifestyle, genetic factors are also one of the main causes of DDH. Certain chromosomes, genes, loci, and polymorphisms are being associated with variable severity of this disorder (Stefan et al. 2020). To reveal the background of DDH heredity, multiple studies focused on large sample sizes with an emphasis on the correlation between genotype, phenotype, and continuous clinical examination (38). By sequencing and analyzing the blood DNA samples of DDH patients, the researchers found some genes may be the pathogenic genes of DDH (Mabuchi et al. 2006; Feldman et al. 2014). In our biobank, the acquisition of 1490 blood, 2172 tissue specimens and 892 DNA samples will greatly contribute to research and a better understanding of disease pathogenesis, genetic polymorphisms. Therefore, many cross-institutional specimens and data from wider regional areas in China not only meet the sufficiency and diversity of samples, but also ensure the reliability of the research results.

It should be noted that our DDH biobank still has some limitations. Considering the incidence characteristics of DDH, in addition to blood and tissue samples, the inclusion of patients' feces in the sample bank project is helpful to study the relationship between intestinal flora and eating habits and the incidence. Even if the specimen of our biobank includes wider areas of China, increasing communication of samples and data may expose patient privacy to the risk of disclosure, and stronger confidentiality protection will be required.

Collectively, we have established a biobank, the first and largest DDH biobank publicly reported in China, with biological specimen and clinical data, to facilitate clinical and basic scientific research. The establishment of China Standardization Organization database is still in its infancy, we believe our work will be conducive to standardized DDH biobank establishment.

## Data Availability

The datasets generated during and/or analyzed during the current study are available from the corresponding author on reasonable request.
